# Compressive Optic Neuropathy Secondary to Sinonasal Undifferentiated Carcinoma in a Young Male

**DOI:** 10.7759/cureus.19042

**Published:** 2021-10-25

**Authors:** Maizatul Nadia Hassan, Wan Hazabbah Wan Hitam, Nurul Ain Masnon, Subash Govindasamy, Ahmad Razif Omar

**Affiliations:** 1 Department of Ophthalmology and Visual Science, School of Medical Sciences, Universiti Sains Malaysia, Kubang Kerian, MYS; 2 Department of Ophthalmology, 96 Malaysian Armed Forces Hospital, Royal Malaysian Navy Base, Lumut, MYS

**Keywords:** young adult male, optic neuropathy, blurring vision, compressive optic neuropathy, sinonasal undifferentiated carcinoma

## Abstract

Sinonasal undifferentiated carcinoma (SNUC) is an extremely aggressive malignancy. Extension to the orbit and adjacent structures is common, but isolated visual loss as a presenting symptom is rare. We report a rare case of SNUC with bilateral visual loss as the initial manifestation. A 34-year-old gentleman was presented with acute onset loss of vision in both eyes for one week. It was followed by recurrent headaches and epistaxis. Visual acuity in the right eye was 2/60 and 3/60 in the left eye. Funduscopy showed a bilateral swollen disc. Neuroimaging revealed a large mass in the ethmoidal sinus extended laterally causing compression to recti muscles and the optic nerves. The histopathological examination of nasal tissue biopsy showed features of SNUC with bone and perineural invasion. A diagnosis of SNUC with bilateral compressive optic neuropathy was established. The patient underwent tumor debulking and base of skull reconstruction by the neurosurgical team. This was then followed by chemotherapy and radiotherapy. The patient’s right eye visual acuity initially improved to 6/9. However, his both eye vision developed into no light perception during treatment. In conclusion, SNUC is a highly aggressive tumor that may present with acute blindness. Early treatment may save a life, but the visual prognosis is guarded due to extensive optic nerve damage caused by tumor compression.

## Introduction

Carcinomas of the nasal cavity and paranasal sinuses (PNS) account for 0.2%-0.8% of all malignant neoplasms [1]. Sinonasal undifferentiated carcinoma (SNUC) is a distinctive carcinoma of uncertain histogenesis composed of pleomorphic tumor cells with frequent necrosis [2]. SNUC is highly aggressive, which is commonly presented as a rapidly enlarging tumor mass involving multiple PNS and nasal cavities with extension to other contiguous sites [3]. Patients with SNUC usually experience acute sinus symptoms, nasal obstruction, facial pain, and epistaxis followed by proptosis, periorbital swelling, diplopia, and symptoms of cranial nerve involvement [2,3]. It is a rare occurrence to have acute visual loss as a presenting symptom. To date, there are only two cases of SNUC that had acute visual loss as an initial presentation of SNUC reported [4,5]. We report a case of SNUC with bilateral acute visual loss as the initial presentation of SNUC.

## Case presentation

A 34-year-old gentleman was presented with acute loss of vision in both eyes and headache for one week. It was later followed by a few episodes of epistaxis. The patient had no nasal symptoms like anosmia, nasal congestion, or obstruction. There were also no ocular symptoms like proptosis, periorbital swelling, or diplopia. He did not have any fever, nausea, vomiting, or loss of weight and appetite. He was a non-smoker and had no family history of malignancy. He worked as navy personnel and denied any exposure to coal, coal, chromium, and nickel.

On examination, the patient’s visual acuity in the right eye was 2/60 and 3/60 in the left eye. Optic nerve functions such as light brightness, red saturation, and color vision tests were reduced in both eyes. There were no limitations of extraocular muscle movement with no diplopia and proptosis. Fundoscopy showed a bilateral swollen disc with a normal macula reflex. As the patient had concomitant epistaxis, a rhinologist was consulted. The nasal-endoscopy assessment showed a polypoidal fleshy mass occupying both nasal cavities, obstructing the osteomeatal complex. Other cranial nerves and central nervous system examinations were normal. Systemic examination was unremarkable.

**Figure 1:**
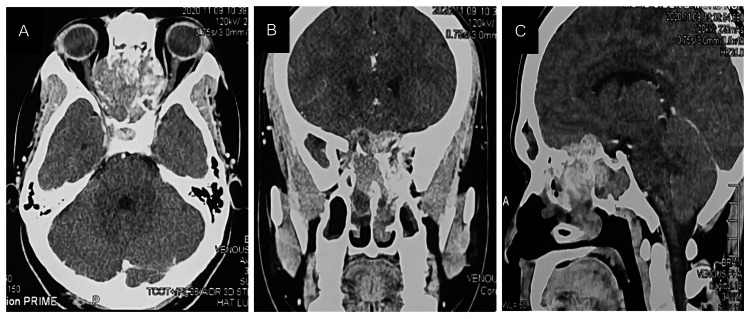
Contrast-enhanced computed tomography of the brain and paranasal sinus (A) Axial CT showed a huge and highly aggressive mass in the ethmoidal sinuses extending to the extraconal compartments of both orbits with compression of both optic nerves and medial recti muscles. (B) Coronal view showed a lesion with bony erosion and osteolysis of the ethmoid sinus, bilateral medial and superior orbital walls including the nasal septum, bilateral superior and middle nasal turbinates as well as bilateral osteomeatal complexes. (C) Sagittal view showed a lesion with bony erosion of the anterior base of the skull and sphenoid sinus with intracranial extension.

**Figure 2:**
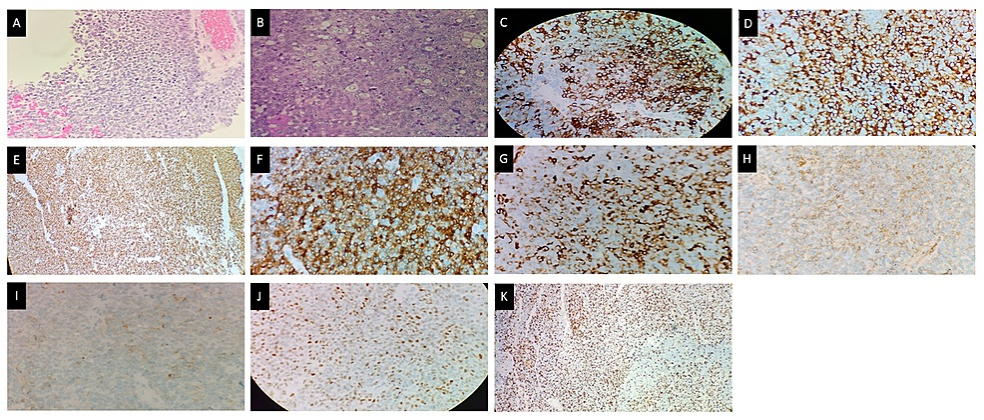
Histopathology and immunohistochemistry assessment of the mass (A) Sections show fragments of tumour tissue composed of infiltrating malignant tumour arranged in trabeculae, lobules, and sheets, with adjacent bone tissue with bony trabeculae and its marrow also infiltrated by the tumour. (B) The tumour cells are moderate to markedly pleomorphic with enlarged round to polyhedral basophilic vesicular nuclei with inconspicuous to prominent nucleoli and scanty eosinophilic cytoplasm. Immunohistochemistery showed the tumour stained positive for (C) cytokeratin 7, (D) epithelial membrane antigen, (E) CKAB1/AE3, (F) BCL-2, (G) vimentin, (H) CD99, (I) synaptophysin, (J) p53, and (K) Ki67 >70% of tumour cells.

Contrast-enhanced computed tomography (CECT) of the brain and PNS revealed a large lobular extra-axial lesion measuring 4.2 x 4.7 x 3.4 cm in the ethmoidal sinus with surrounding bony erosion. The mass extended to the extraconal compartments of both orbits with compression of both optic nerves and medial recti muscles, nasal cavity, sphenoid sinuses, and anterior base of the skull (Figure 1). Biopsy of the mass revealed SNUC with bone and perineural invasion. Immunohistochemistry showed tumor cells were positive for CKAB1/AE3, cytokeratin 7 (CK7), epithelial membrane antigen (EMA), vimentin, CD99, BCL-2, p53, synaptophysin, and Ki67 (Figure 2).

**Figure 3:**
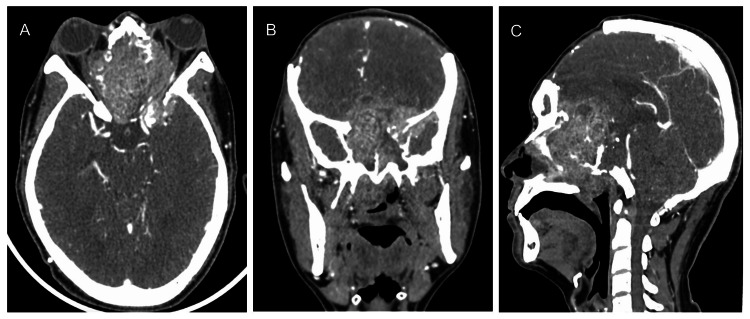
Contrast-enhanced computed tomography of the brain and paranasal sinus (A) Axial CT showed a significantly larger mass in the paranasal sinuses with left eye proptosis and intracranial extension to the bilateral temporal lobes. The mass was invading into intraconal compartments of both orbits with compression and infiltration of both optic nerves. (B) Coronal view showed evidence of bifrontal craniectomy with erosion of bilateral lamina papyracea and involvement of bilateral orbital apex. (C) Sagittal view showed a lesion with bony erosion of the walls of sinuses and clivus with intracranial extension.

The patient underwent successful combined endoscopic and transcranial excision of the tumor. Debulking of the tumor was performed via bicoronal craniectomy with the base of skull reconstruction. Post-operative right eye vision improved to 6/9 but his left eye worsened to no light perception. Funduscopy showed resolving optic disc swelling in both eyes. He was planning for four cycles of chemotherapy. Intravenous cisplatin 75 mg/m^2^ and 5-fluorouracil 750 mg/m^2^ on day 1 to day 5 of adjuvant were given for a total of three cycles. Just before his third cycle of chemotherapy, he complained of worsening right eye vision. CECT of the brain and orbit showed a significantly increasing size of the paranasal mass measuring 6.2 x 5.1 x 7.1 cm. The mass was invading the intraconal spaces bilaterally with compression to both optic nerves (Figure 3). Subsequently, he underwent intensity-modulated radiation therapy (IMRT) of the fasciocervical region with a dose of 70 Gy for 35 cycles for seven weeks and weekly intravenous cisplatin 40 mg/m^2^. During his recent follow-up, his visual acuity remained non-perceptive of light in both eyes with left eye proptosis. Fundoscopy showed bilateral optic atrophy. There was no other neurological problem.

## Discussion

Sinonasal undifferentiated carcinoma is a rare entity specific to the nasal cavities and paranasal sinus. It makes up <1% of all malignancies [6]. A study by Chambers et al. showed that the median age of its occurrence is the sixth decade of life with a prevalence of 24.5% and incidence in younger patients accounts for 1.3%-12.6% of the overall cases [7]. Male predominance was found with a male-female ratio of 2-3:1 [2]. Our patient was a male in his third decade of life when he was diagnosed with SNUC.

The exact pathogenesis of SNUC is unknown. However, SNUC often originates from the Schneiderian epithelium, which lines the nasal cavity and the PNS [8]. About 55% of sinus tumors arise from the maxillary sinus, 35% from the nasal cavity, 9% from the ethmoid sinus, and the rest from the frontal and sphenoid cavity [1]. In our case, the tumor originated from the ethmoid sinus that is an uncommon area. SNUC is also associated with smok­ing, a history of radiation therapy for nasopharyngeal carcinoma, and occupational exposure to coal, chromium, and nickel [2,6]. In some studies, the Epstein-Barr virus has been suggested to be an etiological factor [9,10].

The most common presenting symptoms of patients with SNUC include nasal obstruction, epistaxis, and facial pain, which were described in the previous studies [11,12]. A study by Xu et al. showed 85% of patients were diagnosed with Stage IV SNUC during presentation due to symptoms similar to benign sinus disease [13]. Rarely, patients with SNUC experience visual impairment, periorbital swelling, cranial nerve palsies, or features of distant metastasis such as spinal cord compression as the initial presenting features of the disease [1,6,13]. In our case, despite the local invasiveness of the tumor, there were no sinonasal symptoms except epistaxis, which appeared only after the onset of visual loss due to compression of the bilateral optic nerve. This is a rare presentation of SNUC with only two similar cases that have been reported previously [4,5].

Differential diagnoses of bilateral optic disc swelling in young patients include infections, inflammatory, demyelinating, and neoplasm [14]. In our patient, the CECT showed a huge mass causing compressive optic neuropathy. The list of differentials includes squamous cell carcinoma, olfactory neuroblastoma, neuroendocrine carcinoma, lymphoma, melanoma, rhabdomyosarcoma, lymphoepithelioma, as well as SNUC [15]. Imaging cannot reliably differentiate SNUC from these other entities, and so the diagnosis of SNUC heavily relies on histopathological evaluation, including immunohistochemistry assessment [3]. SNUC shows the pathological characteristics of medium-sized cells in nests or sheets with elevated mitosis rates, tumor necrosis, and vascular permeation. Immunohistochemical analysis of SNUC showed simple epithelial type cytokeratin (CK7, CK8, CK17) and EMA with variable reactivity towards neuro-specific enolase (NSE), tumor marker p53, chromogranin, and synaptophysin. This test is very helpful to discern SNUC from other undifferentiated malignancies, such as olfactory neuroblastoma and undifferentiated nasopharyngeal carcinoma [11].

SNUC is a rapidly growing and locally destructive lesion, which tends to extend orbitally and intracranially beyond usual anatomical limitations of the nasal cavity and PNS. Intensive multimodal treatment, including surgical resection and adjuvant therapy such as radiotherapy and chemotherapy treatment, is required in the treatment of SNUC. Surgery can be done preceding or after chemoradiation [16]. Radical craniofacial resection may be required with maxillectomy and orbital exenteration. Craniofacial resection with a tumor-free margin is positively related to tumor-free survival status [17]. However, SNUC may not be completely treated by surgery or radiation therapy and it has a high rate of both local-regional recurrence and distant metastases [3].

The overall five-year survival for SNUC is estimated at 22% and five-year distant metastasis-free survival is estimated at 35% [18]. Some studies have reported visual improvement after surgery and chemoradiation treatment in a patient with compressive optic neuropathy in SNUC, but this did not happen with our patient [4,5]. His visual acuity remained poor, and his general condition remained good with the ability to carry out daily activities independently. This is similar to previous findings where the mean survival of patients may improve up to 53.6 months with the use of an aggressive multimodality approach [15,19].

## Conclusions

SNUC may occur in young patients and usually presents with the advanced stage of the disease. Infiltration or direct compression of the optic nerve by the tumor may lead to a poor visual prognosis. Combined management by ophthalmology, rhinology, and neurosurgery teams is important. Although prognosis is guarded, timely treatment may halt disease progression, improve the survival rate, and reduce the risk of distant metastasis.
